# Evidence for small-scale torsional Alfvén waves in the solar corona

**DOI:** 10.1038/s41550-025-02690-9

**Published:** 2025-10-24

**Authors:** R. J. Morton, Y. Gao, E. Tajfirouze, H. Tian, T. Van Doorsselaere, T. A. Schad

**Affiliations:** 1 School of Engineering, Physics and Mathematics, Northumbria University, Newcastle, UK; 2 School of Earth and Space Sciences, Peking University, Beijing, China; 3 Centre for mathematical Plasma Astrophysics, Department of Mathematics, KU Leuven, Leuven, Belgium; 4 Department of Physics and Astronomy, Queen Mary University of London, London, UK; 5 State Key Laboratory of Solar Activity and Space Weather, National Space Science Center, Chinese Academy of Sciences, Beijing, China; 6 National Solar Observatory, Makawao, HI USA

**Keywords:** Solar physics, Astrophysical plasmas

## Abstract

The corona is the outermost layer of the Sun’s atmosphere. Its plasma is accelerated and flows out into interplanetary space as a heated, supersonic wind. The details of energy and momentum transfer to the plasma remain debated. Alfvén waves are a favoured mechanism, and in a plasma composed of inhomogeneous flux tubes, the only pure Alfvén mode is torsional in nature. Large-scale modes have been observed sporadically, but a prevalent, small-scale counterpart in the corona has yet to be established. The Daniel K. Inouye Solar Telescope has begun to provide unprecedented views of the Sun, with the Cryo-NIRSP instrument delivering coronal observations with a high spatial and spectral resolution. Here the data reveal that the quiescent corona supports torsional Alfvén waves, which continuously twist the magnetic field lines back and forth. The measured wave amplitudes are small but are probably underestimated due to the line-of-sight integration. The results indicate that the waves may carry a substantial fraction of the energy required to power the quiet Sun and solar wind.

## Abstract (web summary)

This study uses observations from the DKI Solar Telescope to reveal that the Sun’s corona hosts small-scale torsional Alfvén waves. These twisting motions likely carry enough energy to help heat the Sun’s atmosphere and drive the solar wind.

## Main

The Sun’s atmosphere is heated to millions of degrees, which generates X-ray and extreme-ultraviolet radiation that impacts the dynamics and evolution of planetary atmospheres^1,2^. Heated plasma is also accelerated away from the Sun as solar wind, leading to angular momentum loss^3^ and carrying with it transient events that lead to sudden changes in the state of planetary magnetospheres. The mechanisms behind the heating and acceleration are poorly constrained but potentially related. One long-standing candidate is Alfvén waves, incompressible perturbations of the magnetic field capable of transferring energy from the convective motions in the photosphere out into the heliosphere. Numerous Alfvén wave turbulence models can self-consistently reproduce extreme-ultraviolet emission^4^ and solar wind conditions^5–7^, reinforcing the importance of Alfvén waves in energy transfer. Alfvén waves may also be the source of the so-called magnetic switchbacks^7^, which carry Poynting flux into the solar wind^8^.

The solar atmosphere is a highly inhomogeneous plasma. At relatively small scales (100–1,000 km), the corona is composed of compact flux tubes, defined by local, field-aligned density enhancements occurring across a relatively uniform magnetic field. There are indications that the apparent flux tubes may be projections of more complex, diffuse structuring^9,10^. Either way, such structuring leads to a richer spectrum of wave modes beyond those arising from a homogeneous plasma (that is, slow, fast and Alfvén)^11^, with many modes having mixed properties. Of particular interest here are the torsional Alfvén mode, which is the only pure Alfvén mode in a plasma comprising inhomogeneous flux tubes, and the non-axisymmetric kink mode, a mode often referred to as Alfvénic^12,13^.

The presence of the kink mode throughout the corona is well established^14–19^, both from the transverse motions of density structures in imaging observations and the coherent fluctuations of Doppler velocities in spectroscopic data. Kink modes oscillate coherently with neighbouring structures over a range of spatial scales^20^ as well as individually. Estimates of the energy flux of coronal kink waves are debated, with some indicating that the waves carry sufficient energy to power the quiet corona and fast solar wind (>200 W m^−2^)^15^, whereas others indicate that the waves carry only around half that amount^16,17,21^ or even less, depending on the degree of volume-filling by the wave field^22,23^.

Early detections of Alfvén waves in the corona^14,24^ have been contested and identified as the kink mode^25,26^. Reports of excess (non-thermal) coronal spectral linewidths have also often been attributed to the presence of unresolved Alfvén waves^27,28^, although these could also be explained by kink motions^29^. Less controversial evidence for Alfvén modes in the corona has been sporadic and limited to excitation during transient events^30,31^.

This is in juxtaposition with observations of the lower solar atmosphere, with evidence for swirling or vortex drivers arising naturally in the intergranular lanes of the photospheric convection^32,33^. The swirls have been observed co-spatially in the chromosphere with scales of 2–4 Mm (refs. ^34,35^) and can be described as torsional Alfvénic pulses coherent across several flux tubes^36–38^. Possible signatures of torsional Alfvén waves have also been identified in the lower solar atmosphere in individual structures^39–43^. Although the upward wave energy flux to the corona may be reduced by substantial reflection at the transition region^44,45^ (which contributes to localized heating within the chromosphere^42^), different Alfvén modes are expected to coexist in the corona^38,45,46^. Interestingly, the presence of both kink and Alfvén modes can also act in concert to increase the efficiency of energy dissipation through turbulence and phase-mixing^47^.

Using the unique capabilities of the Cryogenic Near-infrared Spectropolarimeter (Cryo-NIRSP)^48^, an instrument of the US National Science Foundation’s Daniel K. Inouye Solar Telescope (DKIST)^49^, we demonstrate that small-scale torsional motions are prevalent in the corona and present alongside the kink motion.

## Results

DKIST is a 4-m-aperture telescope and can provide the highest-resolution views of the Sun. Data were obtained with Cryo-NIRSP during the commissioning phase of the telescope on 30 October 2023 with the express aim of exploiting the high spatial, temporal and spectral resolution to examine wave behaviour in the corona (details are given in Methods).

**Fig. 1:**
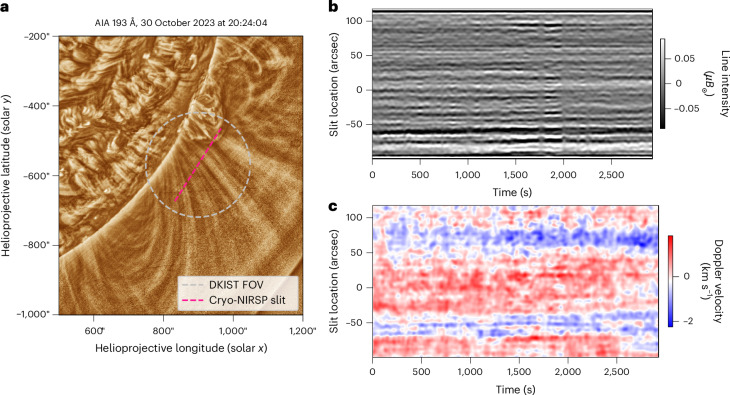
Overview of observations taken on 30 October 2023. **a**, Image of the solar corona taken with the Atmospheric Imaging Assembly onboard the Solar Dynamics Observatory in the extreme ultraviolet (193 Å channel) to provide context for the Cryo-NIRSP data. The DKIST field of view is indicated by the circle, and the approximate location of the Cryo-NIRSP slit is indicated by the red dashed line. **b**, Butterworth-filtered and unsharp masked line intensity data. The data reveal the fine-scale coronal structures that are subject to transverse displacements. Note that variations in seeing quality are visible as vertical bands. **c**, Doppler velocities from the sit-and-stare data (with the temporal mean subtracted from each time series). AIA, Atmospheric Imaging Assembly; FOV, field of view.

The data used here are a spectroscopic sit-and-stare observation of the iron Xiii emission line at 1,074.7 nm, with the Cryo-NIRSP slit held at a fixed location (with a stability of ~0.1″) in the corona at a height of ~0.1*R*_☉_ (Fig. 1). The region observed by the slit covers an open-field region (located at the centre) and crosses quiet-Sun loops at the base of a streamer (and an active region; Fig. 1a and ref. ^50^). Modelling the coronal emission line provides estimates for the line intensity (measured in millions of the solar disk intensity, *μB*_☉_, where *μ* = 10^−^^6^ and *B*_☉_ is the disk brightness) and Doppler velocity^50,51^. Filtering the line intensity by removing the diffuse coronal emission (Methods) reveals the fine-scale coronal structure (Fig. 1b). The excess brightness of the visible flux-tube structures is probably due to an increased density along the line of sight (LOS) compared with the neighbouring regions. A study of the filtered line intensity image (Fig. 1b) reveals that the fine-scale structure is swaying, which is an indicator of the kink mode.

The Doppler velocity signal for the iron Xiii line (Fig. 1c) represents motions along the LOS. Owing to the optically thin nature of the corona, the emergent intensity contains contributions from plasma along the LOS. These contributions will be weighted to the plasma with the largest emissivity, which typically occurs above the limb in the quiet Sun but scales with density. As such, the Doppler velocity is also emissivity weighted along the LOS (see the discussion in Supplementary Information)^52^.

There are probably many different components (for example, flows, waves and turbulence) along the LOS that contribute to the observed Doppler velocity. However, the data are dominated by incompressible fluctuations across a range of temporal scales^50^. Previous observations have established that kink modes are one such wave motion that contributes to the Doppler velocity^14,17–19,53^. Hence, the kink mode is not confined to the plane of the sky (POS) and will have a random polarization with respect to the magnetic field axis. This means that a considered treatment of the kink waves is required to unveil the presence of the torsional motions within the Doppler velocity data.

### Signatures of kink motions

**Fig. 2:**
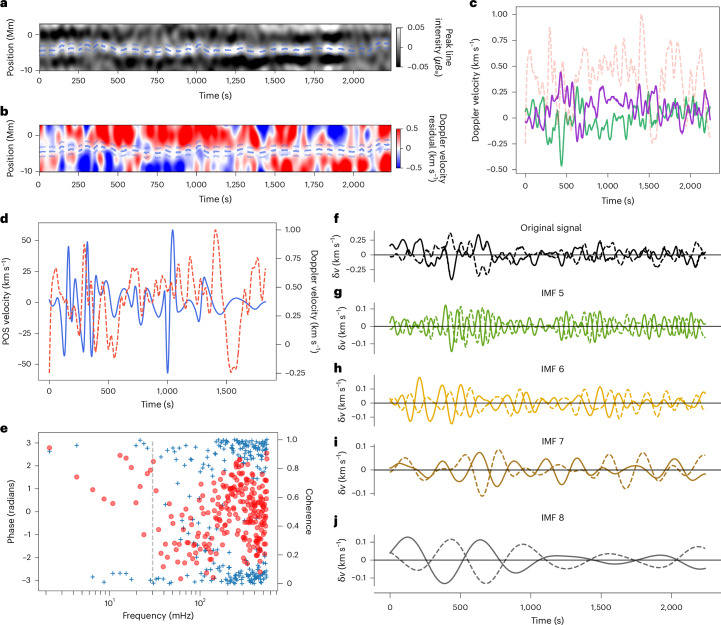
Measurements of coronal waves. **a**, A small region of the peak line intensity focusing on an individual brightness enhancement corresponding to an overdense coronal magnetic flux tube. The thick dashed line (middle) represents the measured centre of the structure as a function of time, which reveals consistent displacements that are a signature of the kink mode. **b**, Residual Doppler velocity after subtracting the Doppler velocity measured at the centre of the flux tube. Patterns of out-of-phase Doppler velocities on either side of the centre of the structure are evident, which is a signature of the torsional motion. **c**, The Doppler velocity at the centre of the flux tube (orange dashed line) and the residual Doppler velocities at a distance of ±1,300 km from the centre of the flux tube, taken from regions marked by the thin dashed lines in **b**. The green solid line is the upper edge. The purple solid line is the lower edge. **d**, POS velocity (blue line) for the kink modes derived from the displacement time series and the Doppler velocity from the centre of the flux tube (orange dashed line). **e**, Cross-spectral analysis of the upper-edge and lower-edge signals. The phase information (blue crosses) shows a consistent ±π out-of-phase relation across many frequencies, and the two signals are coherent (red dots). The vertical grey dashed line indicates the frequency at which the low-pass filtering starts to influence the signals. **f**, Mean-subtracted upper-edge (solid) and lower-edge (dashed) signals. **g**–**j**, Corresponding IMFs derived from EMD: IMF 5 (**g**), IMF 6 (**h**), IMF 7 (**i**) and IMF 8 (**j**).

An example of the analysis of the coronal Alfvénic waves is given in Fig. 2, which focuses on both the line intensity (Fig. 2a) and Doppler velocities (Fig. 2b). The POS motions of the fine-scale structure in the line intensity data are determined by measuring the central location of the flux tubes^21^, thereby obtaining displacement time series (for example, Fig. 2a). From these time series, the wave properties obtained are characteristic of the prevalent, small-scale kink modes found throughout the quiescent corona^15–19,54^, with the typical velocity amplitudes of the order of 15–20 km s^−1^.

**Fig. 3:**
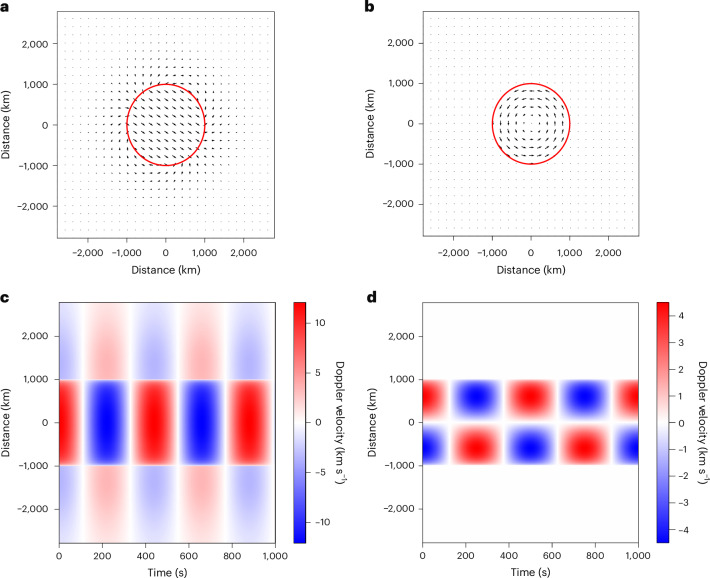
Theoretical predictions of wave signals. **a**,**b**, Velocity vectors calculated from an analytic wave model for the kink mode (*n* = 1) (**a**) and Alfvén mode (*n* = 0) (**b**). The red circle in each panel indicates the boundary of an overdense flux tube in an ambient plasma. **c**,**d**, Corresponding LOS integrated velocities for each mode, which is representative of the Doppler velocity: kink mode (**c**) and Alfvén mode (**d**). The integration is performed over the vertical axis in **a** and **b**. In **a**, the motion of the kink mode is in a plane that is oriented at 35° to the LOS. The LOS Doppler velocity does not depend on the viewing angle for the *n* = 0 mode. Note that the velocity amplitude for each mode is 20 km s^−1^.

**Fig. 4:**
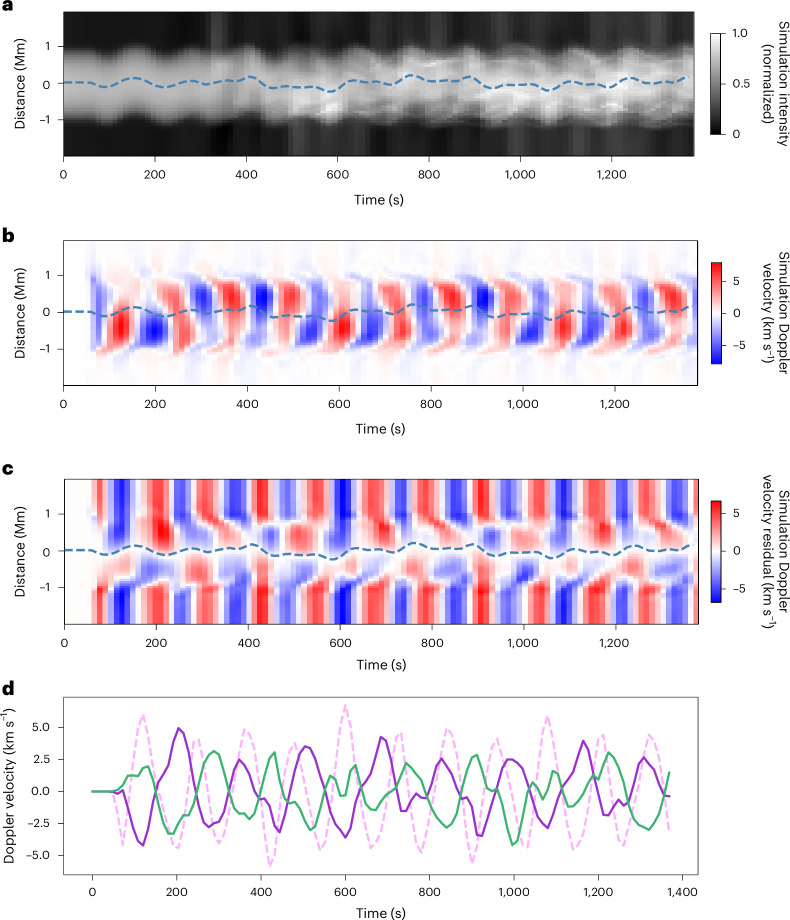
Results of 3D MHD simulations of wave propagation along an overdense open coronal waveguide. **a**, Forward modelled 1,074-nm emission from a flux tube supporting both propagating kink and torsional Alfvén waves. The emission is shown from an LOS that is 45° to the plane of motion of the kink mode. **b**, Corresponding Doppler velocity, which shows a pattern dominated by the kink motions. **c**, Residual Doppler velocity, estimated in the same manner as the observations. Within the flux tube, the pattern of an *n* = 0 torsional Alfvén mode is clearly visible. **d**, Doppler velocities of an individual kink (pink dashed line) and torsional modes (lower edge in purple and upper edge in green). In this simulation of a single flux tube, the amplitudes are larger than the corresponding observed values, which are thought to result from averaging wave motions from several flux tubes along the LOS. The blue dashed lines in **a**–**c** indicate the measured centre of the flux tube.

The kink mode is characterized by the bulk displacement of the density enhancement, but it also perturbs the surrounding plasma. The LOS-integrated velocity (left-hand panels in Fig. 3) from an analytical model for the kink motions (Methods) indicates that the contribution to the Doppler velocity is dominated by the bulk motion. By contrast, the torsional Alfvén modes (with azimuthal wavenumber *n* = 0; right-hand panels of Fig. 3) are dominated by the contributions from the azimuthal velocity component (*v*_*θ*_). Hence, the Doppler velocity along the centre of the flux tube will always be zero or small. This picture is confirmed by a three-dimensional (3D) magnetohydrodynamic (MHD) model^55^ of an oscillating coronal flux tube (Methods), which is driven to excite both the *n* = 0 torsional mode and the kink mode (Fig. 4). The model can account for resonant energy transfer^56,57^, phase-mixing^58^ and instabilities^59,60^ associated with kink motions. The bulk movement of the kink mode still dominates the signal at the centre of the flux tube, with the small-scale dynamics becoming apparent at the boundaries of the flux tube (Fig. 4a,b). Hence, measuring the Doppler velocity at the centre of the flux tube enables an estimate of the kink mode contribution to the Doppler velocity signal (even for several flux tubes along the LOS; see ‘Monte Carlo simulations’ described in Methods and the further discussion in Supplementary Information).

The kink mode estimate is obtained by extracting the Doppler velocity signal at the measured location of the centre of the flux tube (Fig. 2c). A comparison of the Doppler velocity signal and POS kink mode velocity is shown in Fig. 2d. The amplitudes of the POS motion are substantially larger than the amplitudes of Doppler velocity fluctuations. However, a reduction in the Doppler velocities is a well-known issue associated with LOS integration of the radiation through the optically thin corona^29,61^. The correspondence between the POS and LOS motions is supported by the two measures having power spectra with nearly identical shapes.

### Signatures of torsional motions

To reveal any other motions contributing to the Doppler velocity signal, the Doppler velocity signal of the kink mode is removed from a local region around the flux tube to obtain residual Doppler velocities (note that the residual is potentially valid only within the flux tube; Methods). Given the theoretical considerations, the residual signal is assumed to be due to dynamics other than the bulk motion of the kink mode. The residual signal contains oppositely directed Doppler velocities on either side of the flux-tube centre (for example, Fig. 2b). Figure 2c displays the residual Doppler signals sampled on either side of the flux tube, at a distance ±1,300 km from the central location. There is evidence for long-term trends in the residuals, but on short timescales, it is clearly seen that the two residual Doppler velocities (labelled Edge U. and Edge L.) are often in anti-phase. This relation is confirmed through a cross-spectral analysis (Methods), which found a ±π phase difference at frequencies with high coherence between the two residual Doppler velocity time series (Fig. 2e).

As indicated by the wave modelling (Figs. 3d and 4), this Doppler signal is indicative of a torsional motion of the flux tube. For direct comparison, Fig. 4c displays the residual Doppler velocity from the simulations, which shows the same signature of the torsional waves. Figure 4d displays the residual Doppler velocities at the edges of the simulated flux tubes for direct comparison with Fig. 2c.

**Extended Data Fig. 1:**
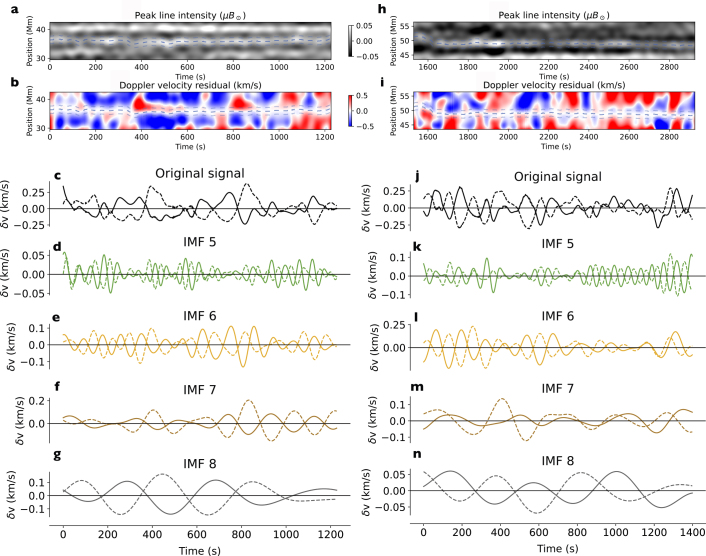
Two additional individual examples of torsional Alfvén motions. Similar to Fig. 2. Panel **a** (**h**) shows a small region of the peak line intensity focusing on an individual brightness enhancement corresponding to an over dense coronal magnetic flux tube. The thick dashed line (middle) represents the measured centre of the structure as a function of time, the two fainter dashed lines either side represent the two edges of flux tube (Edge U. and Edge L.). Panel **b** (**i**) shows the residual Doppler velocity after subtracting the Doppler velocity measured at the centre of the flux tube. Panel **c** (**j**) indicates the mean-subtracted Edge U. (solid) and Edge L. (dashed signals) residual Doppler signals, while panels **d–g** (**k**–**n**) show the corresponding Intrinsic Mode Functions (IMF) derived from Empirical mode decomposition.

**Extended Data Fig. 2:**
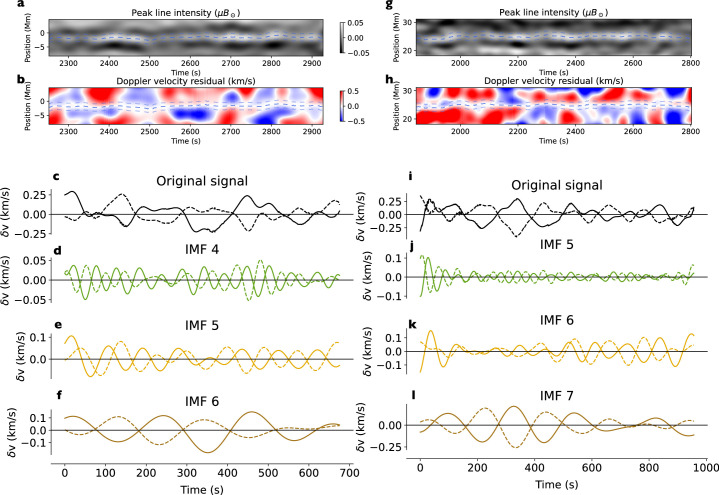
Two additional individual examples of torsional Alfvén motions. Panel **a** (**g**) shows a small region of the peak line intensity focusing on an individual brightness enhancement corresponding to an over dense coronal magnetic flux tube. The thick dashed line (middle) represents the measured centre of the structure as a function of time, the two fainter dashed lines either side represent the two edges of flux tube (Edge U. and Edge L.). Panel **b** (**h**) shows the residual Doppler velocity after subtracting the Doppler velocity measured at the centre of the flux tube. Panel **c** (**i**) indicates the mean-subtracted Edge U. (solid) and Edge L. (dashed signals) residual Doppler signals, while panels **d–f** (**j**–**l**) show the corresponding Intrinsic Mode Functions (IMF) derived from Empirical mode decomposition.

The red/blue asymmetry may not be unique to torsional modes and could arise from other dynamics along the LOS (see ‘Other sources of red and blue asymmetry’ section in Methods). Further confirmation may come from measuring the propagation speeds of the Doppler signals. However, this is not possible with the current slit-based data. Hence, here we apply some additional constraints on the signals to increase the confidence that they arise from torsional Alfvén waves. To be classed as a wave, the signals should have the following properties: (1) They should have more than one cycle with a consistent timescale. (2) There should be similar amplitudes on either side of the flux tube. (3) The zero crossings of the residual Doppler velocity signals on either side of the flux tube should occur nearly simultaneously (indicative of anti-phase behaviour). These features would occur only rarely for other motions along the LOS. To facilitate the comparison, we use empirical mode decomposition (EMD), as it can isolate signals at characteristic timescales from the broadband residual Doppler velocities. EMD separates the signal into so-called intrinsic mode functions (IMFs), which enables identification of potential wave packets. Figure 2f–j shows a typical result of this process. There are fluctuations present in each IMF that meet the above criteria, for example at 600–800 s and 1,400–1,600 s in Fig. 2g. Other examples show torsional Alfvén wave packets with different timescales appearing at the same time (for example, at 200–400 s in Extended Data Fig. 1e,f and at 400–600 s in Extended Data Fig. 2d,e). This behaviour is consistent with that found previously for the small-scale kink motions^21^.

Further analysis of the residual Doppler velocity indicates that the torsional motions seem to be largely confined to the individual flux tubes (Supplementary Discussion and Supplementary Fig. 5). In some instances, the observed torsional signals are pulse-like and seem to extend beyond the flux tube (for example, Fig. 2a at ~200 s). We have not investigated whether all such signals are genuine; they may be a signature of the collective torsional motion of several flux tubes associated with swirls^37,38^. In addition, there are occasions when a torsion-like signal apparently extends beyond 5 Mm from the flux tube (for example, at 700–900 s in Fig. 2a). We discuss several potential explanations for these in Methods.

**Fig. 5:**
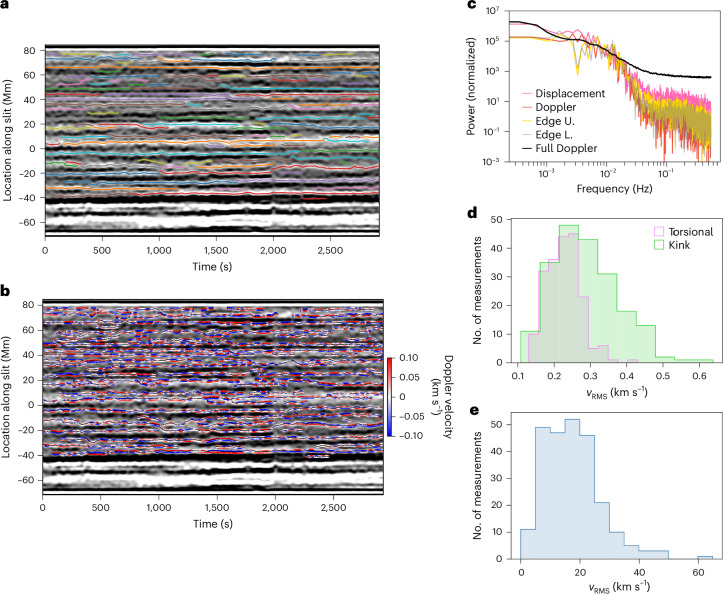
All kink and torsional motions. **a**, Full unsharp masked line amplitude data with the traced coronal structures highlighted by the coloured lines. Each line shows the swaying of the coronal structures, which is due to the kink mode. **b**, Patches of the residual Doppler velocity signal overplotted in their location on the line amplitude data. The regions shown extend ±1,300 km from the central location of each flux tube. The Doppler velocities are clipped to the indicated range for visualization. An examination of the residual Doppler velocity signal highlights many individual examples of torsional motions due to the Alfvén wave. Note we have not shown signals from the region of the slit associated with the active region. **c**, Power spectra for the kink mode displacement (pink) and Doppler velocity (red) signals, the torsional Alfvén fluctuations (upper edge in yellow and lower edge in grey), and the unfiltered Doppler velocity data (black). **d**, Histograms of the r.m.s. Doppler velocities for torsional Alfvén fluctuations (pink) and kink fluctuations (green). **e**, Histogram of r.m.s. velocity amplitudes estimated from the POS motion of the kink mode. The r.m.s. values are calculated for time series of different lengths, with an average number of 400 data points (smallest is 40 and largest is 3,200). Edge L., lower edge; Edge U., upper edge.

**Extended Data Fig. 3:**
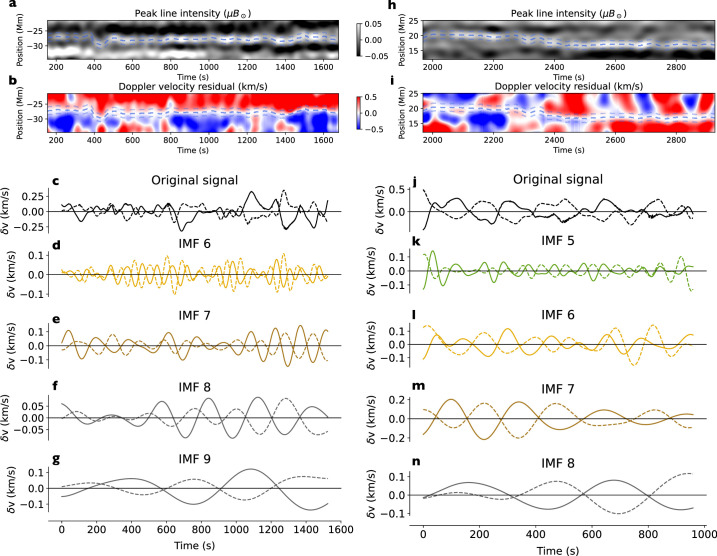
Two additional individual examples of torsional Alfvén motions. Panel **a** (**h**) shows a small region of the peak line intensity focusing on an individual brightness enhancement corresponding to an over dense coronal magnetic flux tube. The thick dashed line (middle) represents the measured centre of the structure as a function of time, the two fainter dashed lines either side represent the two edges of flux tube (Edge U. and Edge L.). Panel **b** (**i**) shows the residual Doppler velocity after subtracting the Doppler velocity measured at the centre of the flux tube. Panel **c** (**j**) indicates the mean-subtracted Edge U. (solid) and Edge L. (dashed signals) residual Doppler signals, while panels **d–g** (**k**–**n**) show the corresponding Intrinsic Mode Functions (IMF) derived from Empirical mode decomposition.

The red/blue anti-phase signals are found throughout the time versus distance Doppler velocity data. By calculating residual Doppler velocities associated with all bright enhancements in the line intensity map from the open field and quiescent region (Fig. 5a), we found many clear examples of red versus blue anti-phase motions (Fig. 5b). Analysis of the individual cases using cross-spectral analysis and mode decomposition supports this (Extended Data Figs. 1–3).

### Wave properties

The torsional motions display a range of timescales but cannot be considered periodic in nature. It is rare to find fluctuations with more than two or three cycles of a similar timescale. Much like the kink modes in the quiet Sun and coronal holes and the Alfvénic fluctuations observed in the solar wind, the torsional fluctuations seem to be broadband and stochastic in nature. In fact, the power spectrum for the transverse displacements, kink-related Doppler velocity and torsional-related Doppler velocity signals all exhibit the same shape (Fig. 5c). This is to be expected, as torsional modes are also thought to be driven by the convective motions of the photosphere, although with different flow patterns (vortices versus buffeting) on different spatial scales (intergranular versus granular).

**Extended Data Fig. 4:**
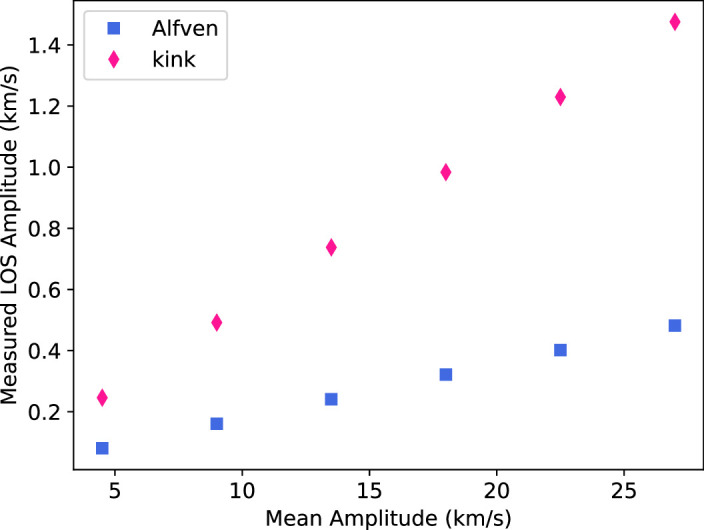
Results from Monte Carlo simulations assessing the impact of line-of-sight integration on the measurement of wave amplitudes. The horizontal axis shows the mean wave amplitude (values drawn from a log-Normal distribution) that represents the true wave amplitudes. The vertical axis is the root mean square Doppler velocity measured after integration along the line-of-sight. The blue squares correspond to the values for torsional Alfvén waves, and the pink diamonds are for the kink mode.

**Extended Data Fig. 5:**
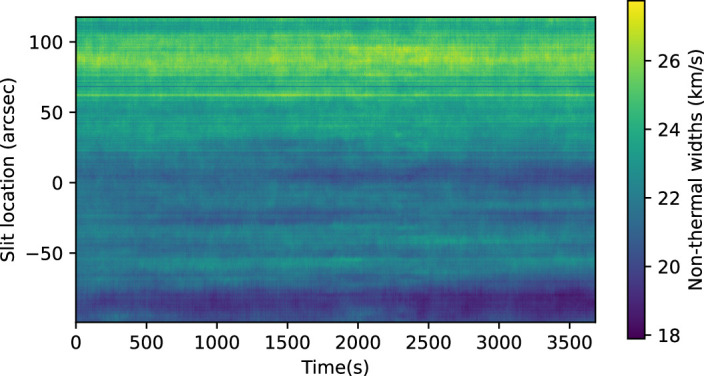
Non-thermal line widths estimated from the 1074.7 nm (Iron Xiii) coronal spectral profiles for the DKIST Cryo-NIRSP data. The figure shows the non-thermal line widths across the slit as a function of time and represent the unresolved motion along the line of sight. The estimated values are in line with the Alfvénic wave amplitudes measured and estimated from the resolved wave motions. The methodology for estimating the non-thermal line widths is discussed in the Methods section.

The distributions of the root-mean-square (r.m.s.) wave velocity amplitudes are shown Fig. 5d,e. As noted, the Doppler velocity values associated with the kink motion are notably smaller than the POS velocities, a factor of ~60 smaller. The mean amplitudes are *v*_LOS,Kink_ = 0.404 ± 0.009 km s^−1^ and *v*_POS,Kink_ = 24.8 ± 0.9 km s^−1^ (both multiplied by factor of $\surd 2$ to convert from *v*_RMS_). Assuming that the reduction in Doppler velocity amplitudes is due to LOS integration effects, then it is conservative to assume the same degree of devaluation for the torsional Alfvén modes. Monte Carlo simulations indicate that the correction factor for torsional Alfvén waves may be larger than that for kink waves (Methods and Extended Data Fig. 4). The mean velocity amplitude for the torsional Alfvén modes is *v*_LOS,Alf_ = 0.321 ± 0.005 km s^−1^, which is slightly smaller than that for the kink modes. This value is larger than the individual mode amplitudes inferred from the EMD analysis (~0.1 km s^−1^) but is consistent with the total energy of a signal containing fluctuations with a broadband frequency spectrum (Parseval’s theorem). After correcting for LOS averaging, the implied mean wave amplitudes are *v*_Alf_ ≈ 19.5 km s^−1^. This value is consistent with the amplitudes previously reported for chromospheric spicules^41,42^. The superposition of out-of-phase fluctuations leads to non-thermal line broadening, and estimates of non-thermal linewidths also provide comparable values (18–30 km s^−1^; Extended Data Fig. 5 and Methods).

## Discussion

The identification of prevalent Alfvén waves in the corona ends a protracted search for these modes, which had its origins back in the 1940s^62^. We anticipate the discovery will be the genesis of further investigations into the propagation and dissipation of the torsional Alfvén waves in the corona, which will be enabled by the ability of Cryo-NIRSP to provide high-quality spectra. Our understanding of Alfvén wave propagation in the solar atmosphere has been hampered by the past inability to measure them directly, so that our knowledge of them has relied heavily on theoretical models. Hence, the detection of Alfvén waves is an essential step for evaluating the physics within the suite of current Alfvén-wave-driven turbulence models.

Although the field of view is small, around 200″, the Cryo-NIRSP slit overlaps diverse magnetic topologies. The torsional motions are evident across the slit, which indicates that their presence is probably independent of the magnetic structure in the corona. Given the known ubiquity of kink modes across the corona, this would imply that Alfvénic wave motions of all types are potentially also prevalent.

For the torsional modes to be of real interest, they need to make a meaningful contribution to the transport of energy. The kinetic energy flux of Alfvénic modes is given approximately by *F* ≈ *ρv*^2^*v*_gr_, where *v*_gr_ is the group velocity, *v* is the velocity amplitude and *ρ* is the mass density. The magnitudes of the Doppler velocities indicate that torsional Alfvén modes have smaller amplitudes than the kink modes do. We suggest that the Alfvén wave amplitudes adjusted for LOS averaging are potentially still an underestimate. First, the Monte Carlo simulations indicate that the LOS Doppler velocities from torsional modes should be a factor of two smaller than those for the kink modes (Methods and Extended Data Fig. 4). Second, the resolving ability of Cryo-NIRSP is seeing-limited to ~0.6″ along the slit, which means that torsional modes with a spatial structure smaller than the resolution will necessarily be averaged to zero due to the axisymmetric nature of the eigenfunctions. Further work is required to quantify the magnitude of this effect, although a conservative estimate indicates that both modes are able to transport the same amount of energy (the group velocity is similar for both modes, which is approximately the Alfvén speed in weakly overdense flux tubes). This agrees with estimates of the energy flux obtained from non-thermal widths (Extended Data Fig. 5) and in previous studies^27,28,63,64^, which are thought to represent unresolved Alfvénic waves. Given the range of past estimates for the energy flux associated with the kink wave^15–17^, this places a conservative estimate for the combined energy flux of Alfvénic (kink and Alfvén) waves between 100 W m^−2^ and 400 W m^−2^, which would be sufficient to explain the heating of the quiet-Sun plasma and the acceleration of the fast solar wind.

## Methods

### Data description

The data were obtained on 30 October 2023 by the Cryo-NIRSP instrument located at the US National Science Foundation’s DKIST. Cryo-NIRSP can observe the corona with a spatial sampling of ~0.12″ (~87 km) along the slit and with temporal cadences of 1 s. The Cryo-NIRSP data used here are a sit-and-stare observation using the 0.5″-wide spectrograph slit with 0.12″ spatial sampling along the slit. The spatial resolution of Cryo-NIRSP is optical-limited to around 0.3″ and seems to be seeing-limited to 0.6″ (due to terrestrial atmospheric seeing)^50^.

The Cryo-NIRSP instrument was tuned to examine the 1,074.7 nm (iron Xiii) coronal emission line and provided high-quality spectra with a spectral dispersion of 4.4 × 10^−12^ m per pixel and a resolving power of *R* *≈* 48,000. The solar spectrum was observed between 1,072 nm and 1,076 nm. This line has been extensively used to probe coronal dynamics^14,17–19,53^ and is resolved well by Cryo-NIRSP^51^. Supplementary Figs. 1 and 2 contain an example spectrum and the corresponding fits to the line profile, along with a discussion on the measurement uncertainty associated with the Doppler velocities.

The target region for the observations was centred on (*X*, *Y*) *=* (898″, −568″) (a height of 0.1*R*_☉_ above the limb). The pointing stability of the data was assessed by examining the motions of the fine structure, and it seems to be ≲0.1″. Hence, the stability of the pointing was better than the estimated seeing resolution along the slit. Further information about the dataset can be found in ref. ^50^.

### Data processing

High-frequency noise in the data can lead to issues when identifying the fine-scale coronal structures. The noise arises from temporal sources (for example, photon shot noise and variations in seeing) and spatial sources (for example, detector artefacts). Hence, a two-dimensional Butterworth low-pass filter was applied both to line intensity and Doppler velocity data. The results of the filtering are shown in Fig. 1b,c.

For the line intensity data, the noise component has δ*I* < 0.15*μB*_☉_, which was substantially smaller than the coronal emission (*I* < 15*μB*_☉_), although it can be comparable in magnitude to the extra emission from the overdense structures. The associated spatial power spectrum for the unfiltered data shows power-law behaviour down to the noise limit (Supplementary Fig. 4), indicating the presence of multiscale structures within the corona^65,66^. The filtering essentially smooths the signal over small scales. Hence, there is a loss of power for spatial variability below scales of 2 Mm and temporal variability below 30 s. Thus, the processed data restrict measurements to motions of relatively large-scale (compared with the instrument resolution) structures and dynamics.

The Doppler velocity data have an estimated noise level of δ*v* < 0.1 km s^−1^ (see Supplementary Information for a discussion). This value was substantially reduced after the application of the low-pass filter. We note that the power spectra shown in Fig. 5c also indicate the impact of the filtering.

### Analysis of kink motions

The location of the fine-scale structure was traced using the NUWT algorithm^21^, which fits a Gaussian to regions of enhanced brightness (overdense structures), with the potential to provide subpixel accuracy on the physical location of the structures. The analysis was performed with unsharp masked line amplitude data, which had also been high-pass filtered spatially with a boxcar average filter (9 Mm wide). This filtering removed the dominant, large-scale coronal emission and left only the brightness differences on scales smaller than 9 Mm (Fig. 1b). The locations of the peak enhanced brightness are traced in time to provide a time series of the central location of the overdense structures. All traced features are shown in Fig. 5a. POS velocities are calculated from the numerical derivative of the time series.

### Non-thermal linewidths

Non-thermal linewidths for the dataset used here are shown in Extended Data Fig. 5. The non-thermal component of the linewidth *ξ* is estimated using the following expression^67^:

$$\xi =\sqrt{\,\frac{{{\rm{F}}{\rm{W}}{\rm{H}}{\rm{M}}}^{2}-{w}_{{\rm{I}}}^{2}}{4{\rm{l}}{\rm{n}}2{({\lambda }_{0}/c)}^{2}\,}-\left(\frac{2{k}_{{\rm{B}}}T}{M}\right)},$$

where *w*_I_ is the instrumental spectral point-spread function, FWHM is the full-width at half-maximum, *T* is the ion temperature (assumed here to be the peak line formation temperature for iron Xiii, ~1.6 MK), *k*_B_ is the Boltzmann constant and *M* is the ion mass.

### The residual Doppler signal

After estimating the locations of the flux tubes, we extract the Doppler velocity signals at the flux-tube centre. Analytical and numerical modelling indicates that this Doppler signal represents the kink mode contribution to the LOS motion. We note that the Doppler signal could include contributions from any non-torsional motion, although other modes (fluting and rotational modes) have not been identified in coronal structures. The focus here is on isolating any *n* = 0 torsional signals; hence, in the following: (1) We justify why the signal extracted at the flux-tube centre does not contain any signal from potential *n* = 0 modes. (2) We discuss why the residual Doppler signal identified as a torsional motion does not arise from the kink mode or a higher-azimuthal-order Alfvén mode. (3) We discuss alternative dynamics that could lead to signals like those of the torsional mode.

Figure 3 displays the velocity vectors for the kink mode (Fig. 3a) and the torsional Alfvén modes (Fig. 3b for *n* = 0 and Supplementary Fig. 7a for *n* = 1) at their maximum values (with a temporal dependence of e^i*ωt*^, where *ω* = 2π*f* is the angular frequency). The calculations were performed for a magnetized cylinder with radius *R* and a piecewise, discontinuous density profile:

$$\begin{array}{l}\rho (r)=\left\{\begin{array}{l}\begin{array}{cc}{\rho }_{{\rm{i}}}, & \,\,\,\,r\le R,\end{array}\\ \begin{array}{cc}{\rho }_{{\rm{e}}}, & \,\,\,\,r > R.\end{array}\end{array}\right.\,\end{array}$$

where *ρ*_i_ is the internal density and *ρ*_e_ is the external density. The magnetic field is homogeneous. Details of how to calculate the mode amplitude profiles (and, hence, velocity vectors) can be found in Supplementary Information.

For the *n* = 0 torsional mode, no matter which LOS is taken with respect to the flux tube, the integrated velocity is always zero at the centre of the flux tube, as the velocity is always perpendicular to the radial direction (*v*_*θ*_ only). One can crudely forward model the expected Doppler velocity signal by taking 〈*v*_LOS_〉, the mean of the velocity signal along the LOS^68^. The calculation of the mean velocity is weighted with respect to the square of the density to mimic the emission measure weighting in an optically thin plasma (with no photo-excitation). The projected LOS Doppler velocity (Fig. 3d) shows that the *n* = 0 torsional mode has zero velocity at the flux-tube centre, whereas the boundaries alternate from blue to red (a well-known result^69^). For the *n* = 1 Alfvén mode, the motion is also predominantly associated with the azimuthal velocities, although there is a small radial component, which leads to the visible symmetry across the flux-tube axis (Supplementary Fig. 7b).

By contrast, the kink mode has a constant velocity amplitude across the cross section of the cylinder, and the Doppler velocity signature depends on the LOS angle with respect to the direction of motion. If motion is perpendicular to the LOS, there is no Doppler signal. There is no contribution from the internal plasma, and the external velocities cancel out due to symmetry. At other LOS angles relative to the plane of the kink motion, the Doppler velocity profile peaks at the flux-tube centre and is near constant across the cross section. The Doppler velocity signal in the external region is 180° out of phase with the Doppler signal in the internal region and has a much smaller amplitude.

The above discussion assumes that the coronal structures can be modelled as discrete, isolated density enhancements, which is not realistic. Coronal structures are thought to transition smoothly from the internal to external plasma. Alfvén waves are known to propagate along magnetic surfaces. The model used assumes that the coronal structures can be modelled as a magnetized cylinder and constrains the magnetic surfaces to be within the cylinder. However, in the real corona, gradients in the magnetic field and modes are not strictly confined to the visual boundaries^9,10^. In principle, several Alfvén modes could exist if several magnetic surfaces are present^70^, which could explain the apparent extension of the torsional Alfvén modes beyond the visual boundaries of the observed flux tubes (as noted in the main text).

A smoothly varying density profile from the tube interior to the exterior could lead to resonance effects and excite further rotational motions (principally *n* = 1 Alfvén modes) within the region of smoothly varying density^12,13,71^. The presence of these rotational modes in the current observations depends upon the damping length of the kink mode, which is proportional to the density contrast (*ρ*_i_/*ρ*_e_)^72^. In general, kink modes in open-field regions seem to be weakly damped^17^, which is in line with the small density contrasts in coronal holes inferred from imaging observations^66^.

### 3D MHD simulations

To validate the method for extracting torsional wave signals, we performed a 3D MHD simulation, focusing on transverse waves in coronal waveguides with a forward modelling of the expected infrared radiation. The simulation set-up closely follows ref. ^55^, with a stratified, density-enhanced magnetic flux tube oriented perpendicular to the solar surface, which can mimic open coronal structures^73^. The tube has a radius of 1 Mm, with both the tube axis and the background magnetic field (~10 G) aligned along the *z* axis. The density contrast was initially set as *ρ*_i_/*ρ*_e_ = 3, with internal density *ρ*_i_ = 7.5 × 10^−15^ g cm^−3^. We also introduced a boundary layer (width = 0.6 Mm) with a hyperbolic tangent profile. The initial set-up was relaxed for 2,400 s to achieve a nearly magnetostatic state without notable background velocity. The simulation domain spans from [−3, 3] Mm × [−3, 3] Mm × [0, 150] Mm, with a uniform grid of 256 × 256 × 256 cells. The 3D time-dependent ideal MHD equations were solved using the PLUTO code^74^. Further details of the model can be found in ref. ^55^.

A continuous velocity driver was applied at the lower boundary of the flux tube to generate both kink and torsional Alfvén waves (Supplementary Fig. 6), as observed by Cryo-NIRSP. The driver was horizontal (only in the *x–y* plane) and localized in the flux-tube region, so that it acted as a superposition of a kink wave driver (with a period of 300 s) and a torsional Alfvén wave driver (periods varying radially from 150 s to 280 s). The velocity amplitudes for both drivers were set to 8 km s^−1^, and the kink wave driver was aligned along the *x* axis. Further details about the wave driver are provided in Supplementary Information.

The wave driver generated a mixture of kink and torsional Alfvén waves, which, in principle, resembles our observational results. Forward modelling was undertaken with FoMo^75^ to synthesize the iron Xiii 1,074.7-nm spectral profiles for certain LOS angles. We focused on the case when the LOS forms a 45° angle to the *x* axis (direction of the kink driver) and lies in the horizontal (*x–y*) plane. The synthesized spectral profiles were integrated along the LOS to provide two-dimensional distributions of the line intensity and Doppler velocity. Time versus distance maps were constructed at *z* = 50 Mm (Fig. 4a,b).

The time versus distance map for the Doppler velocity primarily exhibits the characteristic of kink waves, with periodic redshifts and blueshifts across the flux tube^55,76^ (see also Fig. 4b). However, when we subtracted the Doppler velocity at the centre position (indicated by the blue dashed line), the residual velocity revealed a typical torsional wave signal (Fig. 4c) that closely resembles Figs. 2b and 3d. In addition, the residual Doppler velocities at the upper and lower edges of the flux tube clearly have an out-of-phase pattern.

The residual Doppler velocity (Fig. 4c) also reveals some artefacts arising from the subtraction methodology. The LOS Doppler velocity profile for the kink mode is not uniform across the flux tube (for example, Fig. 3d). Hence, subtracting the LOS velocity value at the centre of the flux tube still leaves some impression of the kink mode. This leads to locations where the torsional mode signal seems to extend beyond the boundaries of the flux tube (for example, around 200 s, 600 s, 900 s and 1,200 s). There is also a clear impact on the residual Doppler velocity pattern beyond the flux tube where the oscillation is not present. Hence, the residual Doppler velocity signal is potentially valid only for the flux tube upon which it is calculated. However, the kink modes on several neighbouring flux tubes can oscillate coherently^20^, and in such situations, the residual Doppler velocity may be valid beyond the flux tube. Hence, we advise that the residual Doppler velocity signals from beyond the flux-tube boundary should be interpreted with caution.

#### Mode decomposition

The time series for the residual Doppler velocity is made up of fluctuations that span a range of timescales (for example, Fig. 2c), which is captured by the power spectrum of the time series (Fig. 5c). This is expected, as the waves are thought to be excited by solar convection, which is a broadband turbulent driver. A previous analysis of kink modes revealed that fluctuations with a characteristic timescale usually persist for only one to four cycles, and these can appear as wave packets, with several timescales excited simultaneously^21^. This means that Fourier analysis is not necessarily the best tool for studying the fluctuations. Hence, we use EMD^77^, a well-established tool for analysing non-stationary series. Unlike Fourier analysis, there are no predefined basis functions. Instead, the method defines the IMFs adaptively from the data. The IMFs are locally defined to permit a time versus frequency analysis, which contrasts with the global frequency representation in Fourier analysis. We have confirmed that similar results can be obtained using frequency filtering with Fourier methods.

Figure 2f–i and Extended Data Figs. 1–3 show the IMFs derived from the residual Doppler velocities. Only the IMFs associated with the longer timescales are shown because the IMFs associated with shorter timescales contain mainly data noise or have amplitudes ≲0.05 km s^−1^. In some cases, some of the signals with small amplitudes may be genuine. However, we chose to be conservative and focused only on the motions with larger amplitudes.

Note that the IMFs can suffer from mode-mixing, so that a single IMF may contain fluctuations on disparate timescales or similar fluctuations may be split across different IMFs. Mode-mixing is probably an issue in some of the examples shown here, as the power spectrum of fluctuations shows that the signals are broadband and do not have well-separated timescales. Hence, the strict conditions we placed on whether a fluctuation constitutes a torsional mode (main text), such as equal amplitudes and simultaneous zero crossings, could potentially be relaxed.

### Monte Carlo simulations

It is expected from previous work that LOS integration of the radiation through the optically thin corona leads to a reduction in wave amplitudes for the kink modes^29,61^. Here we develop Monte Carlo simulations to show that this is also the case for the torsional Alfvén mode.

It follows from a consideration of optically thin radiation that the level of reduction of the LOS Doppler velocity (from the sum of oscillations with a random phase) will, in principle, be similar for both modes (the derivation is in Supplementary Information). Note that the reduction depends on the volume of ambient plasma compared with the volume of the flux tube. Considering Fig. 3, the LOS averaging of the torsional mode with *v* = 20 km s^−1^ for a single cylinder gives *v*_LOS_ = 6 km s^−1^. By contrast, the LOS averaging of the kink mode with *v* = 20 km s^−1^ for a single cylinder gives *v*_LOS_ *=* 18 km s^−1^ (the case in Fig. 3 incurs a further reduction due to the 35° angle with the LOS). Hence, there is a notable reduction in the torsional Alfvén mode amplitude when integrating over the LOS compared with the kink mode. This is because the direction of the velocity vectors within the flux tube for the torsional mode depends upon the spatial location, whereas for the kink mode, the velocity vectors all point in the same direction. The decrease in the torsional wave amplitude is greater (on average) than the reduction in the kink mode amplitude due to the random polarization angle with respect to the LOS (which leads to a $1/\surd 2$ average decrease, bringing the 18 km s^−1^ down to ~13 km s^−1^).

This was confirmed by a Monte Carlo simulation inspired by methods used previously^78^. We provide a discussion in Supplementary Information as to why we believe this approach provides useful results, while acknowledging the limitations.

Using analytic models of the waves, many (200) oscillating flux tubes were stacked along the LOS, each with a different phase, period and amplitude. The phase and period were drawn from uniform distributions, ${\mathscr{U}}$(0, 2π) and ${\mathscr{U}}(100,\,300)$, respectively. The amplitude was drawn from log-normal distributions^21^, with the mean amplitude varying between 4 km s^−1^ and 30 km s^−1^ for different simulations. The LOS velocity was calculated by integrating the velocities over all the flux tubes and weighting with the emission measure. The emission measure decreased gradually towards and away from the central flux tube, with a decay length of 800 Mm. This was done for the Alfvén and kink modes separately. As anticipated, we found that both modes suffered a substantial reduction in the measured Doppler velocity compared with the actual mode amplitudes (Extended Data Fig. 4). The LOS velocity of the Alfvén mode was roughly half that of the kink mode, in line with the expectations from the single flux tube. The results support our suggestion that actual Alfvén and kink wave amplitudes being equivalent is conservative.

We ran a second Monte Carlo model to investigate how the LOS integration influences the emergent Doppler velocity signal beyond amplitudes. In the simulation, we again stacked several single flux tubes oscillating along the LOS, focusing on only the torsional Alfvén mode. The amplitude and frequency of the torsional Alfvén mode were defined by a power-law spectrum. Each flux tube had its own time series that obeyed the same power law. The Doppler velocity through the flux tubes was averaged along a LOS with a weakly decaying emission measure. This can be considered a worst-case scenario, as the emergent intensity was only weakly weighted to the structure with the largest emission, as many other structures along the LOS have a similar emissivity.

The emergent Doppler signal was a combination of all Doppler signals along the LOS and did not match the Doppler velocity signal at the ‘brightest’ flux tube (Supplementary Fig. 8). The emergent pattern is a superposition of the motions. However, there are three important results from this: (1) The observed Doppler velocities show a pattern of fluctuating red/blue asymmetry across the flux tube. (2) The power spectrum for the LOS Doppler velocity has the same spectral slope as the input spectrum. (3) The power is reduced by a similar magnitude as in previous Monte Carlo simulations. Hence, in the worst case, the amplitude fluctuations and the timescales of the LOS Doppler velocity are still representative of the behaviour for torsional modes on individual flux tubes along the LOS. In reality, the emergent Doppler velocity pattern is probably weighted towards the features visible in the line amplitude data, that is those above the limb. This gives us confidence that the observed signals we see are genuine indications of the torsional motion.

### Other sources of red and blue asymmetry

We also acknowledge other potential dynamics in the corona that could lead to a red/blue asymmetry across the flux tubes. The red/blue asymmetry across the loop could be due to transverse kink motions in individual flux tubes, either in the background or foreground. We expect that several structures will present kink motions along the LOS, and there are two cases to discuss. For kink waves driven by stochastic, uncorrelated drivers, then the amplitudes, periods and phase of two independent oscillations are unlikely to be the same. If the waves are correlated, which can occur over spatial scales of 4–8 Mm (refs. ^20,50^), they will be in phase, so there would be no asymmetric signal. The same reasoning applies to turbulent motions as well.

Another origin of the red/blue asymmetry across the loop is due to a background gradient. One option could be oppositely directed flows from neighbouring loops situated along the LOS, which may occur if heating events are highly localized. The background gradient case can be ruled out if there are several cycles or similar magnitude signals either side of the flux-tube centre.

## Supplementary information

Supplementary Information Supplementary Discussion and Figs. 1–8.

## Data Availability

The DKIST data used for this study are available at: https://nso.edu/dkist/data-center/. The data were recorded on 30 October 2023 using Cryo-NIRSP (dataset ID JAXBIO). The data recorded by the Atmospheric Imaging Assembly onboard the Solar Dynamics Observatory are available at jsoc.stanford.edu.

## Code availability

The code used for the data analysis part of the study is available at https://github.com/Richardjmorton/NUWT and https://github.com/tschad/cryonirsp-notebooks. The open-source MHD simulation code PLUTO^74^ is available at https://plutocode.ph.unito.it/download.html. The FoMo code^75^ is available at https://github.com/TomVeeDee/FoMo.
