# Supplementary material for: Evidence for small-scale torsional Alfvén waves in the solar corona
Source: Nat Astron. 2025 Oct 24;10(1):42–53. doi: 10.1038/s41550-025-02690-9 (PMC12827048; doi:10.1038/s41550-025-02690-9)
Supplement: Supplementary file 1 — Supplementary Discussion and Figs. 1–8. [file 41550_2025_2690_MOESM1_ESM.pdf]

nature astronomy

Supplementary information

https://doi.org/10.1038/s41550-025-02690-9

# Evidence for small-scale torsional Alfvén waves in the solar corona

In the format provided by the authors and unedited

# Table of contents

1. Impact of seeing on kink motions
2. Uncertainty on Doppler motions
3. Spatial and temporal power spectrum
4. Localisation of the residual Doppler velocity signal
5. Emissivity weighting of Doppler velocities
6. Theoretical wave mode profiles
7. A discussion on the Monte Carlo set-up
8. Wave driver in the MHD simulation
9. Supplementary Figures 1-8
10. Supplementary References

**1. Impact of seeing on kink motions**

The main impact of seeing is to degrade the image's spatial resolution; it can do this through image "blur" (i.e. local convolution of a PSF) or via subfield motions (small local tip/tilts of the image). However, on the longer timescales of the total exposure time (~1 sec), we suspect the "blur" term dominates. That said, there can also be image jitter frame-to-frame that can affect the image quality. However, from analysis of the images, the jitter is thought to be on scales less than the blur (<0.6”).

An internal check of the consistency is via the power spectrum. In Morton et al. (2025) we show that the Doppler velocity power spectrum discussed here is the practically the same as that of CoMP (although better resolved). The CoMP instrument has a much smaller aperture (20 cm), so would not be sensitive to small-scale tip-tilt effects. Hence, the motions associated with the Doppler velocity are Alfvénic in nature. The power spectrum of the POS displacements is near-identical (except for a scale factor) to the Doppler velocities associated with the structure (as shown in Figure 5c). This would likely not be the case if the POS displacements were from seeing.

**2. Uncertainty on Doppler velocities**

We provide here an indication of the uncertainties associated with estimates of the Doppler velocities from the fitting to the 1074 nm line. The spectral profiles are fit using a non-linear least-squares method (Schad et al. 2023). The fit is performed without providing errors on the photon counts for the data, which are difficult to estimate due to a complex optical path. This makes it challenging to estimate the uncertainty on parameters from the least-squares method. One method to estimate the errors on the Doppler velocity is to examine the pixel-to-pixel differences, either spatial or temporal (Olsen 1993). The assumption in this approach is that there is little physical variability between neighbouring pixels. This method suggests that the Doppler velocity has an average noise level of ~0.07 km/s.

As an alternative approach, we also fit an example spectrum with a Bayesian version of the model presented in Schad et al. (2024). This is a computationally intensive process, so has not been performed for all spectra in the data set. The priors of the model are not

informative (confirmed with prior predictive checks) so the results should be directly applicable to the least-squares. One benefit of the Bayesian approach is that it enables the inclusion of a free parameter to estimate the variance of data noise in the spectrum (assumed zero mean and normally distributed). We fix this to a constant variance across the spectrum.

The results of the Bayesian fit to the data are shown in Supplementary Figure 1. The top panel shows an example fit to the Cryo-NIRSP spectrum and the resulting Bayesian model. We overplot the posterior mean model and the 95% highest density interval (HDI) for the model posterior. The model for the spectrum is extremely well constrained by the data, as evidenced by the small interval for the HDI.

The lower panel shows the residuals. The residuals are <10% of the radiance compared with the coronal line, $\sim 0.4\ \mu B_{\odot}$. The residuals are dominated by instrumental fringing (Schad et al. 2023). The posterior mean for the Gaussian fit to the coronal line and the 95% HDI are also displayed. The coronal line is extremely well constrained which indicates that the Doppler shifts will also be accurate.

For the shown example, the marginal posterior for the Doppler velocity indicates that an uncertainty of 0.25 km/s. This is a conservative value as the uncertainty will scale with the estimate for the data noise. Here, the data noise in the Bayesian model is effectively estimated from the residuals, which we know are dominated by the fringing.

In Supplementary Figure 2 we also show an example of a spectrogram and the resulting model fit for all spectral profiles along the slit.

We can also get a feeling for the pixel-to-pixel noise in the Doppler velocity from the results themselves. Supplementary Figure 3 shows the unfiltered Doppler velocity results across one of the over-dense features at the peak of a torsional motion (corresponds to the fluctuation seen in Figure 2b around 430s). The small-scale (pixel-to-pixel) variability of the Doppler measurements is at most 0.1 km/s. A clear trend in the Doppler velocity across the structure is evident (structure centre indicated by the vertical line) and is well above the noise.

**3. Spatial and temporal power spectrum**

For reference, in Supplementary Figure 4 we show the spatial and temporal power spectrum before and after the application of the Butterworth filter. The filter suppresses a significant amount of the high spatial-frequency structuring and high temporal-frequency dynamics within the data. For the Doppler velocities, the original signal becomes noise dominated at scales of 0.06 Hz. The application of the filter reduces the power of the noise by close to 94%, assuming the noise is white noise (which has a constant power across all frequencies). Hence, the noise on the Doppler velocities is drastically reduced compared to the values discussed above.

**4. Localisation of the residual Doppler velocity signal**

In Figures 2, ED1, ED2 and ED3, the residual Doppler velocity contains contributions from all spatial and temporal scales. The large-scale patterns can dominate the visual impression in these plots. Careful study of the figures presented in the main paper

reveals the variation in residual Doppler velocity closer to the identified flux tubes is often different from that of the large-scale variation. This indicates the torsional motions are often confined to the visible flux tubes. On occasions, there is correspondence between the extended Doppler velocity pattern and that closer to the flux tube (this could arise from wave drivers coherent over a larger spatial scale, e.g., swirls).

To support the claim of localisation, we present here an additional piece of data analysis to quantitatively define the differences between fluctuations within the flux tube and the external plasma. We only included one example here for brevity, but it is representative.

The analysis undertakes the following steps: i) obtain Doppler velocity at tube centre; ii) extract Doppler velocity profile at an offset $dx$ from the tube centre; iii) subtract centre signal from offset time-series. iv) Calculate the EMD for each residual signal. v) Assign each time-series to a single row to remove the spatial offsets of the Doppler velocity signal arising from the motion of the flux tube. Hence each row represents the Doppler signal at a fixed distance from the features centre. vi) The variation between the EMD signals at different offsets can be quantified by estimating the correlation, the circular correlation (correlation of phase information) and the mean phase differences. The instantaneous phase (obtained from the Hilbert transform) is used to calculate the circular correlation and phase differences. All quantities are calculated with respect to a reference time-series near the tube centre. The mean phase difference has been modified to account for the $\pi$ jump that occurs across the flux tube centre to improve visualisation and interpretability of the figures.

Note, that steps i)-iv) were how the Edge U and L series were analysed.

Supplementary Figure 5 shows the result for the case presented in Figure 2. We note there is usually no discontinuity in wave properties at the flux tubes boundaries, as might be expected from the analytical models (Figures 3 and 4). The corona is not likely to be composed of discrete isolated waveguides. Such structures are likely only theoretical constructs. Additionally, the data is subject to blurring from seeing conditions, which would smooth any small-scale discontinuities (e.g., Guo et al. 2019).

It can be seen visually that the behaviour the residual Doppler signal (in each IMF) changes beyond the inner set of dashed lines (i.e., phase and amplitude variations). Usually, the difference is stark by the outer set of dashed lines. The values of correlation, circular correlation and mean phase differences support this visual impression. Correlation values are typically high close to the flux tube and drop with distance. The phase difference is near zero within the flux tube and increases with distance. Hence, this indicates the torsional motions are largely confined to the visible flux tubs. We note that these three measures are quite coarse as we are averaging over the torsional and non-torsional motions in the residual Doppler velocity.

**5. Emissivity weighting of Doppler velocities**

We can examine how the LOS Doppler shifts will scale with respect to the actual wave amplitude. The intensity for an individual spectral line in an optically thin plasma is given by the emissivity, $\epsilon$, integrated along the line of sight:

$$I(\lambda) = \int \epsilon(\lambda, z)\, dz \ = \int \epsilon(z)\, \phi(\lambda, z)\ dz$$

Where the line profile of the spectral line is assumed Gaussian and given by

$$\phi(\lambda, z) = \frac{1}{\sigma_w \sqrt{2\pi}}\ exp\ \left(-\frac{(\lambda\ - \lambda_D(z))^2}{2\ \sigma_w^2}\right)$$

such that $\int \phi d\lambda = 1$. Here $\sigma_w$ is the thermal width of the line. $\lambda_D(z)$ is the Doppler shift at each plasma element along the LOS.

The first moment is given by the integral over wavelength,

$$I_0 \ = \int \epsilon(z)\ \phi(\lambda, z)\, d\lambda\, dz \ = \int \epsilon(z)\ dz$$

which gives the total intensity of the line. The second moment provides us with an estimate for the Doppler shift, i.e.,

$$\lambda_{D,LOS} \ = \ \frac{1}{I_0}\int\ \epsilon(z)\phi(\lambda, z)\lambda\ d\lambda\, dz \ = \ \frac{1}{I_0}\int\ \epsilon(z)\ \lambda_D(z)\ dz$$

which is seen to be the emissivity-weighted Doppler shift.

The torsional Alfven mode and kink mode are orthogonal solutions to the MHD equations. In the linear regime, they propagate independently and can be linearly superposed, as they do not couple or exchange energy. Hence, for each line profile along the line of sight, the velocity leading to the Doppler shift is the sum of the Alfven and kink contributions

$$\lambda_D \ = \lambda_A \ + \lambda_K$$

which leads to:

$$\lambda_{D,LOS} \ = \frac{1}{I_0}\ \int\ \epsilon(z)\lambda_A dz\ +\ \frac{1}{I_0}\int\ \epsilon(z)\lambda_K\ dz$$

Hence, in principle, if one can estimate the Doppler velocity associated with the kink mode along the line of sight, then it can be subtracted from the measured Doppler velocity to leave the component arising from the torsional Alfven waves along the line of sight.

**6. Theoretical wave mode profiles**

The solutions to the MHD equations provide a description for the wave modes present in a system. Here we use the linear approximation to calculate the mode amplitudes and velocity vectors shown in Figure 3. Details of the process to derive the following equations are given in Spruit (1982), Edwin & Roberts (1983), Goossens et al. (2014).

For the kink mode, we use the specific formalism given in Goossens et al. (2014). The equations for the Lagrangian displacement for kink and fluting modes as a function of radius are:

$$\xi_r\ (r) = A\frac{\kappa_i}{\rho_i\ (\omega^2 - k^2 v_A^2)}B'(x)$$

and

$$\xi_\theta(r) = iA\frac{1}{\rho_i(\omega^2 - k^2 v_A^2)}\frac{n}{r}B(x)$$

where $x = \kappa_i r$ and

$$\kappa_i^2 = \frac{\omega^2 - k^2 v_A^2}{v_A^2}$$

Here, $A$ is a constant, $B(x)$ is the appropriate Bessel function and $'$ indicates the derivative with respect to $x$. There is also the magnetic field $B$, the density $\rho$, the azimuthal mode number, $n$, the frequency, $\omega$, the wavenumber, $k$, and the Alfvén speed, $v_A$.

To model the torsional Alfvén modes, we use the formulation discussed in van Ballegooijen et al. (2011). In general

$$v(r, \theta, 0) = \nabla f \times \hat{z}$$

where $f$ is the stream function. For torsional Alfvén modes this has the form

$$f(r, \theta) = A J_n(a_i\, r) cos\,(n\theta)$$

where $a_i$ is the zero of the Bessel function, $J_n$. Hence,

$$v(r, \theta, 0) = \frac{1}{r}\frac{\partial f}{\partial \theta}\hat{r} - \frac{\partial f}{\partial r}\hat{\theta}$$

**7. A discussion on the Monte Carlo set-up**

There are many open questions to the fine-scale density structure of the coronal holes, meaning that any model will have a significant amount of flexibility due to the large potential parameter space (e.g., spatial structure of density enhancements, level of over density compared to ambient plasmas, separation between sources, number of sources that could contribute, optical path lengths, level of emission measure weighting). Hence, we acknowledge that any Monte Carlo model we use here is likely be a simplification of the true corona in many ways.

We can make some general statements about the fine scale features we find in the data, which supports the approach. There is little evidence for multiple overlapping or crossing bright structures in the open field regions [Morton & Cunningham 2023], a feature more commonly seen in active regions. Similarly, we find little evidence for overlapping structures in the line amplitude data here, that is structures crossing each other in the plane of sky. Amplitudes of swaying motions are small (<500 km) compared to the relative size of structures and the apparent distance between them (~4000 km; [Uritsky et al. 2021; Morton & Cunningham 2024]). This is also the case for the quiescent Sun. In active regions, where loop bundles overlap due to the increased complexity, several observations report loops overlapping during oscillations.

This suggests two things. First, the proximity of neighbouring density enhancements in the open field regions, at least those with significant emissivity, is much further than suggested by the projections onto the plane of sky. Closely packed bright structures would be seen to constantly crossing each other, as reported in active region observations. Secondly, it is likely that the Doppler signals are predominantly associated

with the feature seen in the projection, with contributions from weaker features ahead and behind. If this were not the case, we would observe features moving either into a LOS or emerging from behind an existing bright feature.

However, we do not have knowledge of the spatial scales or geometry of the plasma with largest emissivity along the line of sight. The projection on the POS looks like it could be a compact flux tube, but we are cautious against claiming this to be the case [Malnushenko et al. 2022].

**8. Wave driver in the MHD simulation**

In our MHD simulation, the wave driver is a combination of a kink wave driver and a torsional Alfvén wave driver. The kink wave driver is described by

$$\vec{v}_i(x,y) = v_0 cos\left(\frac{2\pi t}{P}\right)\vec{e}_x,$$
$$\vec{v}_e(x,y) = v_0 R^2 cos\left(\frac{2\pi t}{P}\right)\left[\frac{x^2-y^2}{(x^2+y^2)^2}\vec{e}_x + \frac{2xy}{(x^2+y^2)^2}\vec{e}_y\right],$$

where $\vec{v}_i$ and $\vec{v}_e$ denote the velocity fields inside and outside the flux tube, respectively. To avoid numerical issues at the boundary between the two regions, a transition layer is implemented (Pascoe et al. 2010; Guo et al. 2019). The torsional wave driver, inspired by Guo et al. (2019) and Díaz-Suárez & Soler (2021), has an azimuthal velocity component given by

$$v_\theta = A(r) v_0 sin\left(\frac{2\pi t}{P_A(r)}\right) = A_0 r\ exp\left(-\frac{r^2}{\sigma^2}\right) v_0\ sin\left(\frac{2\pi t}{P_A(r)}\right).$$

Here, the period $P_A(r)$ varying from 150 s (inside the flux tube) to 280 s (outside the flux tube) as a function of $r = \sqrt{x^2+y^2}$ to highlight the non-collective nature of torsional Alfvén waves (see Guo et al. 2019). The $\sigma$ and $A_0$ are set to be 0.8 Mm and 2.9, respectively, to ensure the maximum velocity amplitude can reach $v_0$. With the modulation factor $A(r) = A_0 r exp\left(-\frac{r^2}{\sigma^2}\right)$, the velocity amplitude will peak at $r = 0.6$ Mm and decreasing to 0 when approaching the tube centre and moving outward. The final wave driver is a sum of these two drivers, where $v_0$ is set as 8 km/s. Supplementary Figure 6 shows the variation of $P_A(r)$ and $A(r)$ as functions of $r$.

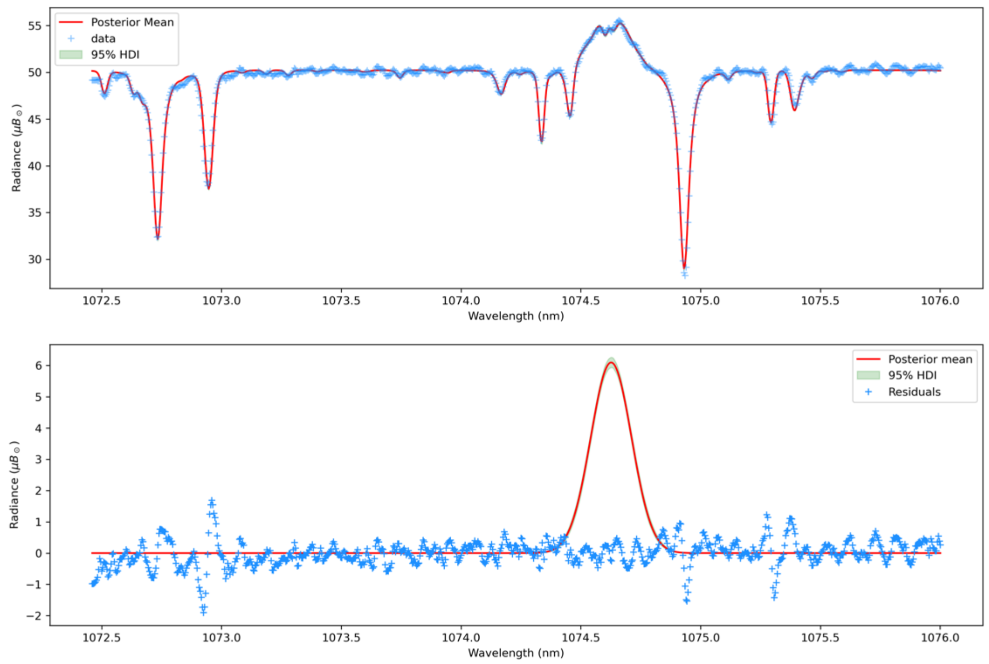


**Supplementary Fig. 1** Results of Bayesian model fit to a Cryo-NIRSP spectrum. The top panel shows the measured signal (blue crosses) along with the posterior mean for the full model (red) and the corresponding 95% highest density interval (HDI – shaded green region). Note that the model is so well constrained by the data that the 95% HDI is so narrow it is difficult to see. The bottom panel shows the model residuals (blue crosses) and the posterior mean model for the Gaussian component that parametrises the coronal line (red). The HDI for this model is shown as the shaded green region.

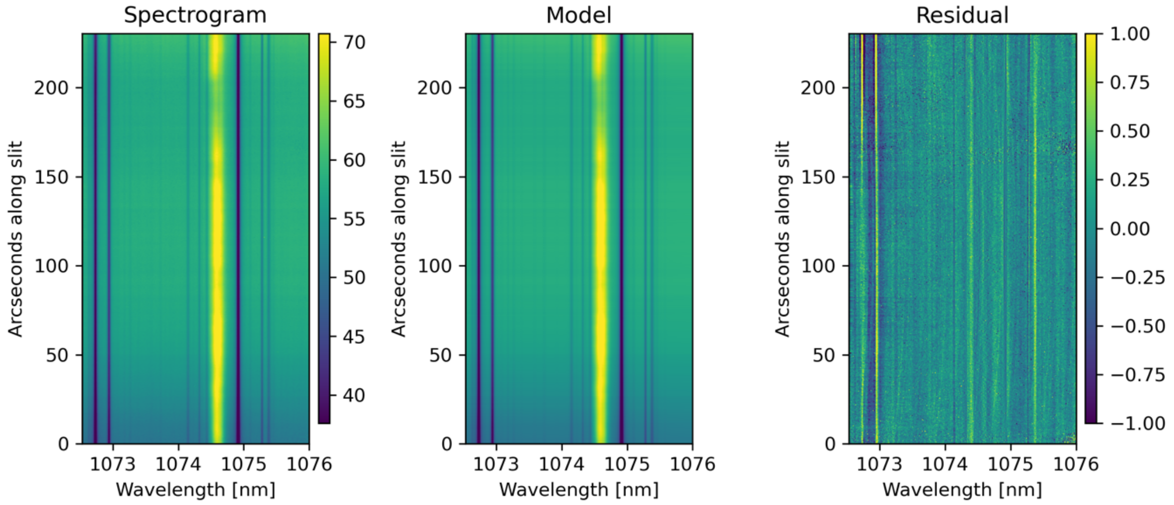


**Supplementary Fig. 2** An example spectrogram is shown in the left-hand panel. The resulting model from the line fitting is shown in the middle panel. The right-hand panel shows the residuals. Colour bar units are in $\mu B_{\odot}$.

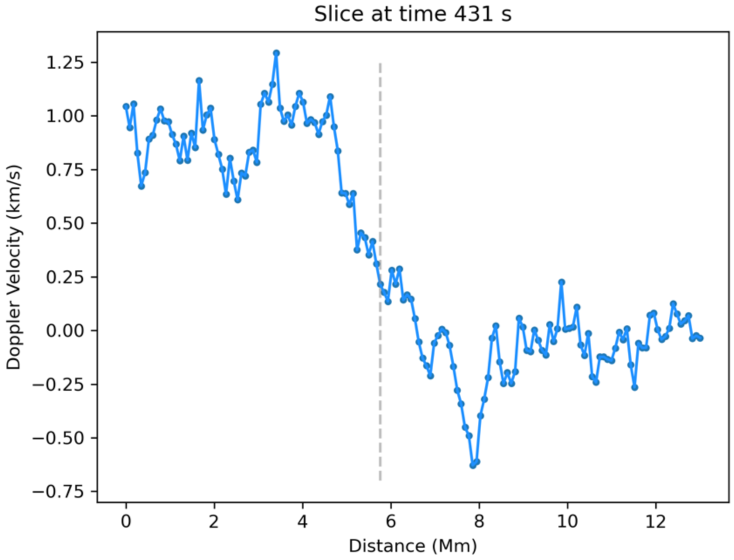


**Supplementary Fig. 3** Example Doppler velocity data from a large-scale torsional motion from the example shown in Figure 2b at 431 s. The data shown is unfiltered, so represents the inclusion of the full noise (i.e., worst case scenario). The dashed grey line is the central location of the fine structure.

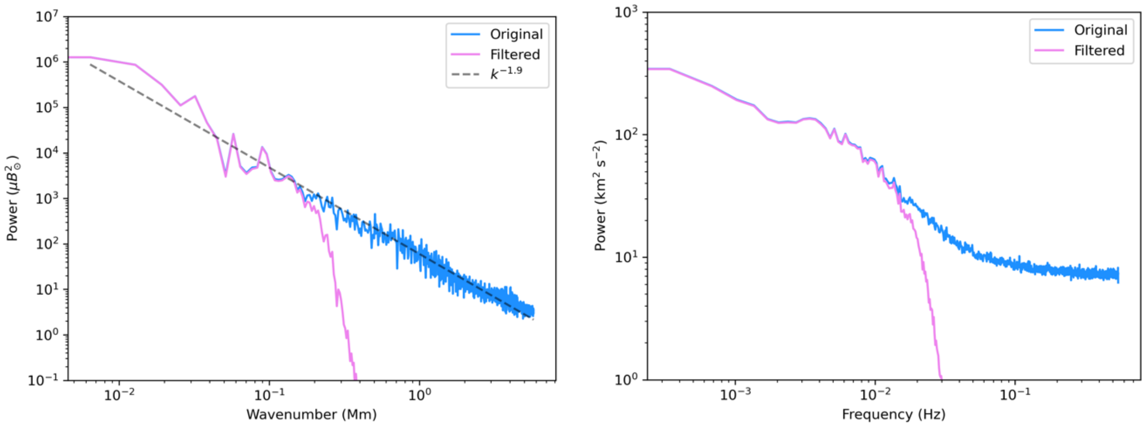


**Supplementary Fig. 4** The left panel shows the spatial power spectrum for the line intensity data (averaged over time) before (blue) and after (violet) the application of the Butterworth filter. A power law is also overplotted for reference. The right panel shows the temporal power spectrum of the Doppler velocity data (averaged along the slit) before and after filtering.

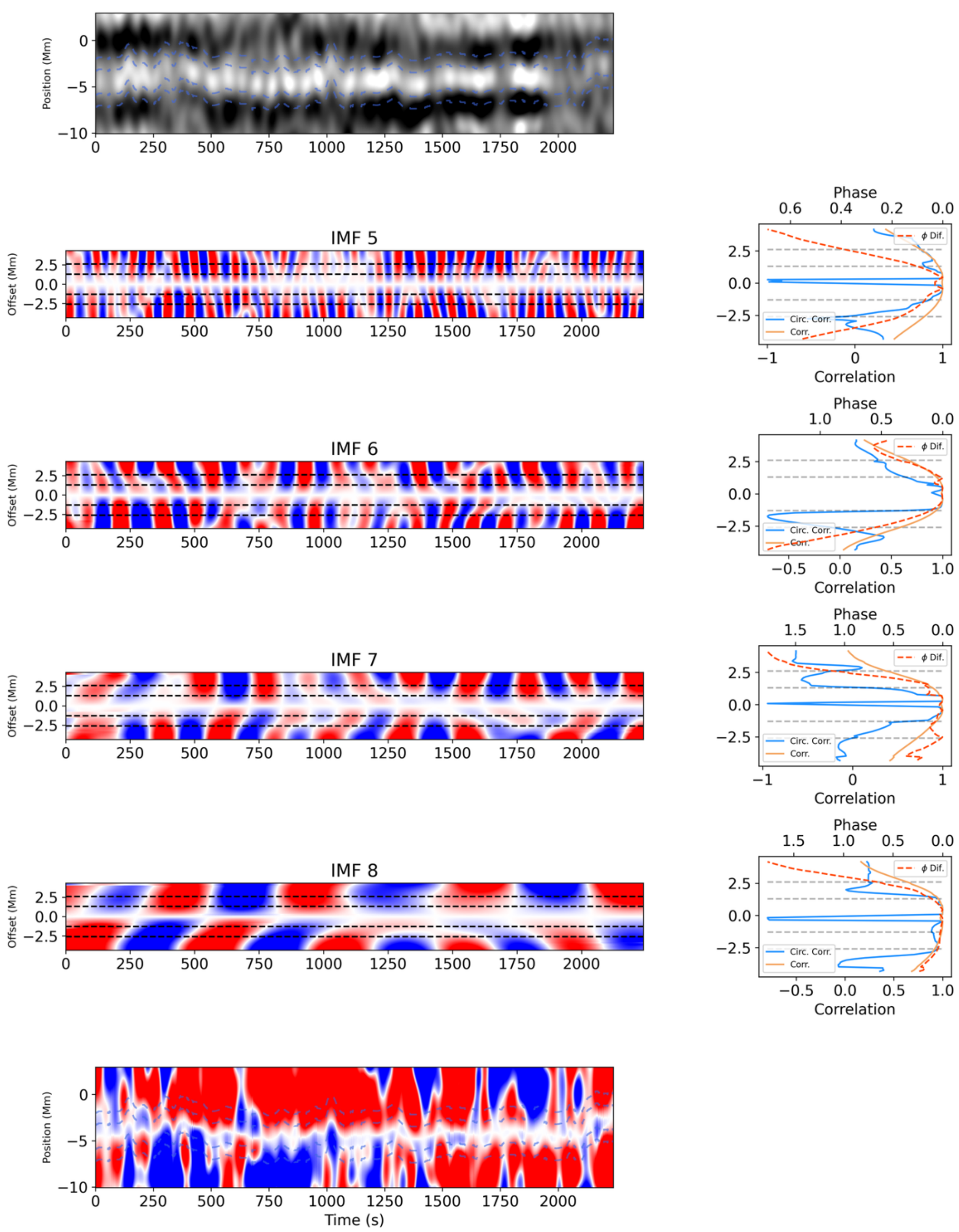


**Supplementary Fig. 5** Examination of localisation of torsional motions corresponding to case in Figure 2. Top panel is line centre intensity. Bottom panel is residual Doppler velocity. The middle panels correspond to the results from supplementary analysis (see Supplementary section 4 for details), showing IMFs 5, 6, 7, 8 from the EMD. Each row in each of these panels represents the IMFs of the Doppler signal at a fixed distance from the flux tubes centre. Inner dashed lines (on all panels) are close to apparent edge. Outer dashed lines (on all panels) are a rough indication of potential neighbouring flux tubes. All Doppler signals are clipped to $\pm 0.1$ km/s for visualisation. The right-hand column displays the correlation (orange), circular correlation (blue), and mean phase difference (red dashed) calculated from the IMF decompositions.

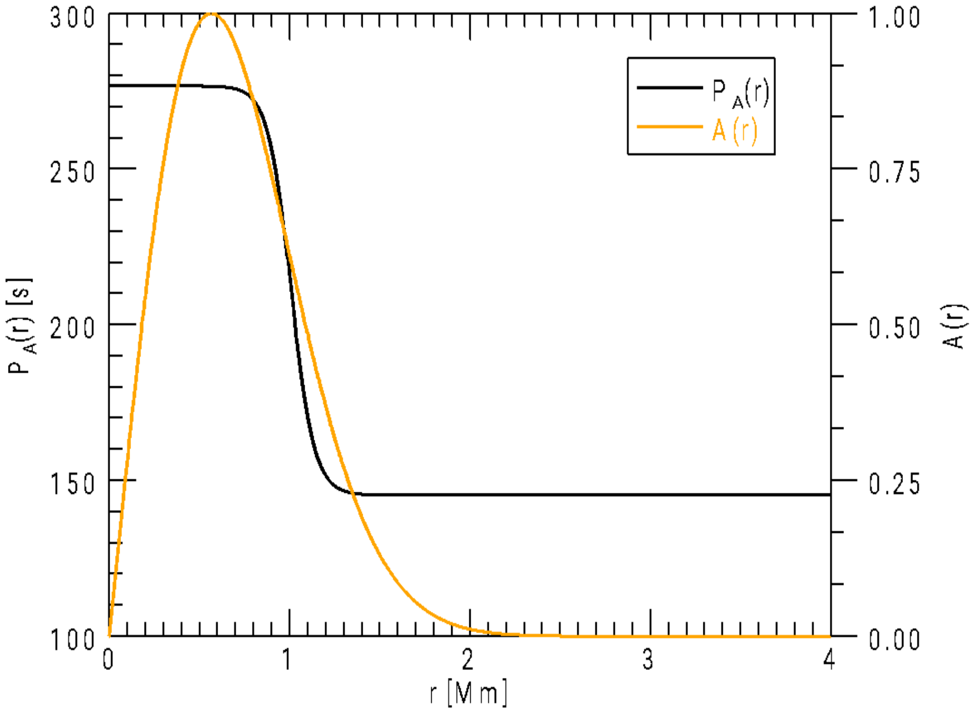


**Supplementary Fig. 6**. Torsional wave driver properties from MHD simulation. The period ($P_A$) and amplitude ($A$) as functions of $r$.

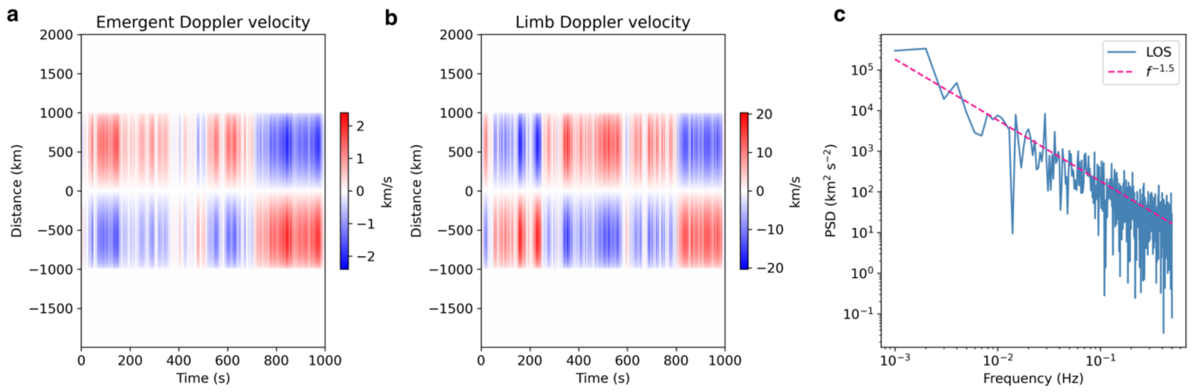


**Supplementary Fig. 7.** Results from a second Monte Carlo simulations of the impact of line-of-sight integration on the appearance and power spectrum of torsional Alfvén waves. Panel a shows the emergent Doppler velocity and panel b shows the Doppler velocity associated with the highest emission flux tube (which we refer to as the limb). In this simulation, it is noticeable that multiple flux tubes along the line of sight contribute nearly equally to the emergent Doppler velocity. Panel c shows the measured power spectrum for the emergent Doppler velocity signal (blue) and the power law (dashed line) from which the motions where drawn (multiplied by a scaling factor to account for the decrease in LOS amplitudes).

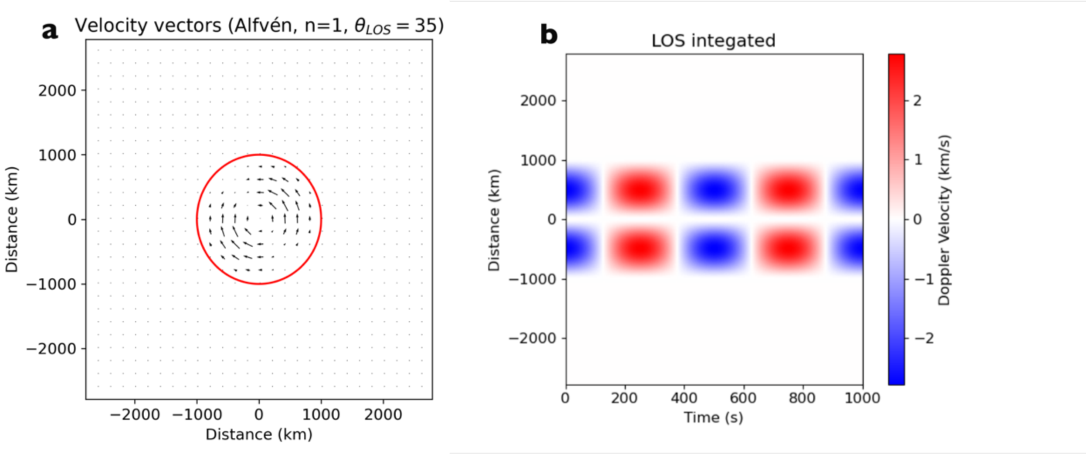


**Supplementary Fig. 8.** Theoretical predictions of Alfvén ($n = 1$) mode Doppler velocity. Panels a show the velocity vectors calculated from an analytic wave model. The angle is with respect to the plane of symmetry. Panel b shows the corresponding line-of-sight integrated velocities, which is representative of the Doppler velocity. The integration is performed over the vertical axis in panels a. Note that the velocity amplitude for the mode is 20 km/s.

**Supplementary References**

1. Schad, T. A. et al. 2023 Astrophysical Journal, 943, 59
2. Olsen, S. I. 1993 CVGIP: Graphical Models and Image Processing, 55, 319
3. Schad, T. A. et al. 2024 Astrophysical Journal, 965, 40
4. Spruit, H. C. 1982 Solar Physics, 75, 3
5. Edwin, P. & Roberts, B. 1983 Solar Physics, 88, 179
6. Goossens, M. et al. 2014 Astrophysical Journal, 788, 9
7. Pascoe, D. J., Wright, A. N., & De Moortel, I. 2010, Astrophysical Journal, 711, 990
8. Guo, M. et al. 2019 Astrophysical Journal, 870, 55
9. Díaz-Suárez, S., & Soler, R. 2021, A&A, 648, A22
